# Composite spheres made of bioengineered spider silk and iron oxide nanoparticles for theranostics applications

**DOI:** 10.1371/journal.pone.0219790

**Published:** 2019-07-15

**Authors:** Kamil Kucharczyk, Jakub Dalibor Rybka, Michael Hilgendorff, Michal Krupinski, Mariusz Slachcinski, Andrzej Mackiewicz, Michael Giersig, Hanna Dams-Kozlowska

**Affiliations:** 1 Chair of Medical Biotechnology, Poznan University of Medical Sciences, Poznan, Poland; 2 Department of Diagnostics and Cancer Immunology, Greater Poland Cancer Centre, Poznan, Poland; 3 Center for Advanced Technology, Adam Mickiewicz University, Poznan, Poland; 4 Institute of Experimental Physics at Freie Universität, Berlin, Germany; 5 The Henryk Niewodniczanski Institute of Nuclear Physics, Polish Academy of Sciences, Krakow, Poland; 6 Faculty of Chemical Technology, Institute of Chemistry and Technical Electrochemistry, Poznan University of Technology, Poznan, Poland; Brandeis University, UNITED STATES

## Abstract

Bioengineered spider silk is a biomaterial that has exquisite mechanical properties, biocompatibility, and biodegradability. Iron oxide nanoparticles can be applied for the detection and analysis of biomolecules, target drug delivery, as MRI contrast agents and as therapeutic agents for hyperthermia-based cancer treatments. In this study, we investigated three bioengineered silks, MS1, MS2 and EMS2, and their potential to form a composite material with magnetic iron oxide nanoparticles (IONPs). The presence of IONPs did not impede the self-assembly properties of MS1, MS2, and EMS2 silks, and spheres formed. The EMS2 spheres had the highest content of IONPs, and the presence of magnetite IONPs in these carriers was confirmed by several methods such as SEM, EDXS, SQUID, MIP-OES and zeta potential measurement. The interaction of EMS2 and IONPs did not modify the superparamagnetic properties of the IONPs, but it influenced the secondary structure of the spheres. The composite particles exhibited a more than two-fold higher loading efficiency for doxorubicin than the plain EMS2 spheres. For both the EMS2 and EMS2/IONP spheres, the drug revealed a pH-dependent release profile with advantageous kinetics for carriers made of the composite material. The composite spheres can be potentially applied for a combined cancer treatment via hyperthermia and drug delivery.

## Introduction

Cancer is one of the major causes of death worldwide. Chemotherapy, which is one of the most common cancer treatments, is often restricted by the resistance of cancer cells to chemotherapeutic drugs [1]. Moreover, due to the low efficiency of the targeted delivery, the anticancer drugs have toxic side effects by causing damage to healthy tissues. Furthermore, resistance to chemotherapeutics or cross-resistance to other drugs can be acquired after cancer therapy, which can lead to recurrence of the disease [2]. The most common reason for the ineffectiveness of chemotherapy is drug efflux from cells through transporting protein [3]. One of the approaches to overcome this phenomenon is extending the blood circulation time of the therapeutic agents to allow their accumulation in the target tissue. The results of the recent research indicated the potential of nanoparticles as a drug delivery platform; they can deposit at the tumor site and deliver a drug to cancer cells [4–6]. In the most studies, nanoparticle accumulation was due to the enhanced permeability and retention (EPR) effect through the leaky vasculature of tumors and the lack of effective lymphatic drainage [7].

Magnetic nanoparticles are a major class of nanomaterials with great potential in various diagnostic and therapeutic applications [8]. Iron oxide (Fe_3_O_4_) nanoparticles have been intensively investigated as magnetic resonance imaging (MRI) contrast agents and as drug delivery carriers [9–12]. Moreover, the superparamagnetic property of IONPs enables their application in hyperthermia treatment [13–15]. Since a temperature above 43°C induces cell death through apoptosis and the sensitivity of tumor cells to an increase in temperature is higher than that of normal cells, hyperthermia therapy can be applied for cancer treatment [16].

Despite the advantages of iron oxide particles, their toxicity and nanotoxicity [17] is one of the main problems for their *in vivo* application. The results of several studies showed that the accumulation of iron ions causes neuronal damage in the brain and inhibits the synthesis of proteins associated with the growth of neural cells [18, 19]. Therefore, IONPs are being combined with various materials to form non-toxic composites. Khandare et al. demonstrated the potential of a magnetic composite composed of carbon nanotubes covered with magnetite nanoparticles, poly(ethylene glycol) (PEG) and fluorophore fluorescein isothiocyanate (FITC) for cellular imaging [20]. To improve magnetic nanoparticle delivery, polymer-like polylactide-co-glycolide (PLGA) was successfully combined with IONPs [21]. The results of Shi et al. demonstrated PEGylated fullerene/iron oxide nanocomposites as a promising tool for melanoma theranostics applications [22]. Nevertheless, the synthetic polymers may have some limitations, e.g. relatively low biocompatibility, biodegradability, and unfavorable byproducts, thus naturally derived materials may provide an alternative tool for composite materials production. Silks are polymers characterized by the remarkable mechanical properties. Their biocompatibility and biodegradability encourage wide biomedical application. Moreover, the recent development of a bioengineered silk production process solved the problem of spider silk accessibility [23]. Silk proteins can be processed into various morphological forms such as fibers, films, hydrogels, scaffolds, micro- and nano-spheres [24]. The potential of silk proteins to form a platform for chemotherapeutics delivery has been investigated [25]. Both silkworm silk nanoparticles and spheres made of bioengineered spider silk bind and release various drugs [5, 26–31]. Moreover, bioengineered spider silk can be functionalized to gain a new function or modify its properties. For instance, genetic engineering of a silk sequence resulted in new silk with an opposite charge [32]. Moreover, genetic modification of silk increased the ability of silk nanoparticles to bind and accumulate inside cells, which improved the efficiency of drug delivery [5, 33].

Recent studies demonstrated the possibility to combine iron oxide nanoparticles and silk fibroin into a composite scaffold. Magnetic silk fibroin scaffolds were not toxic and improve cell adhesion. The scaffolds also exhibited superparamagnetic behavior and excellent hyperthermia properties under exposure to an alternating magnetic field [34]. Deng et al. reported that iron oxide nanoparticles coated with silk fibroin had a higher saturation magnetization than the previously studied PEG-chitosan/Fe_3_O_4_ nanoparticles. The obtained composite particles also exhibited good neural-cytocompatibility [35]. Microspheres, formed from iron oxide and silk fibroin, effectively bound and released doxorubicin and selectively accumulated in the cytoplasm of cancer cells (HeLa) [4]. However, the presented composite spheres were large (up to 3000 nm in diameter) with a wide size distribution, which may be a limiting factor for *in vivo* applications [4]. Another approach for magnetic silk sphere production was examined by Chen et al. where the iron oxide/silk fibroin particles with a more uniform size distribution and mean particle size of 112 nm were prepared by using suspension-enhanced dispersion by supercritical CO_2_. This method of sphere production enabled to obtain iron oxide nanoparticles coated with a silk fibroin protein [36].

In this study, we examined three bioengineered spider silk proteins, MS1, MS2, and EMS2. The proteins were derived from the dragline silk proteins of *Nephila clavipes* spider MaSp1 (MS1) and MaSp2 (MS2 and EMS2) and differ in their properties due to various amino acid compositions [30, 31]. These proteins were blended with iron oxide nanoparticle suspensions and analyzed for their ability to form spheres and bind to IONPs. The EMS2 sphere variant had the highest affinity for IONPs. We investigated the morphology, size, zeta potential, secondary structure, iron oxide content, magnetic properties and cytotoxicity of EMS2/IONPs spheres. Moreover, loading and release studies with chemotherapeutic doxorubicin (Dox) on EMS2/IONP spheres were performed. The obtained results demonstrated the possibility of forming composite spheres made of silk and iron oxide nanoparticles that carry and release drugs. Due to their properties, these particles could have great potential in both magnetic resonance imaging applications and hyperthermia combined with drug delivery therapy against cancer cells.

## Materials and methods

### Construction, production, and purification of bioengineered silk protein

The construction of expression vectors carrying MS1 [37], MS2 [30] and EMS2 [31] was described previously. These expression plasmids were transformed into *E*.*coli* BLR bacteria (DE3) (Novagen, Madison, WI) and the large-scale expression was performed using Bioflo 3000 fermentor (New Brunswick Scientific, Edison, NJ) as previously reported [31].

For protein purification, the thermal denaturation method, named 80/20, was used as described previously [37]. In brief, the bacterial pellet was suspended in a buffer containing 20 mM HEPES (4-(2-hydroxyethyl)-1-piperazineethanesulfonic acid), pH 7.5, 100 mM NaCl, supplemented with Protease Inhibitor Cocktail (2 mM AEBSF, 1 mM phosphoramidon, 130 mM bestatin, 14 mM E-64, 1 mM leupeptin, 0.2 mM aprotinin, and 10 mM pepstatin A; Sigma, St. Louis, MO) and lysed with 0.2 mg/mL lysozyme (Thermo Fisher Scientific, Inc.,Waltham,MA). Then, the lysate was sonicated (3 x 10 s), treated with 0.1 mg/mL DNaseI and 100 mM MgCl_2_ (Sigma, St. Louis, MO) incubated at 4°C for one h and centrifuged at 20 000 x g for 30 min at 4°C. Precipitation of bacterial proteins was performed by heat denaturation (80°C) for 20 min. After centrifugation at 20 000 x g for 30 min, silk protein was precipitated from the supernatant with 20% ammonium sulfate. Subsequently, the silk protein was collected by centrifugation at 10 000 x g for 30 min, rinsed with 20% ammonium sulfate and dissolved in 6 M guanidine thiocyanate. The silk solution was dialyzed against 10 mM Tris-HCl buffer, pH 7.5 using ZelluTrans Dialysis Tubing with an MWCO of 12000−14000 Da (Carl Roth, Karlsruhe, Germany). The protein was concentrated using Amicon tube filters MWCO = 10 kDa (Millipore, Jaffrey, NH) and stored at -80°C until use. The proteins concentration was determined by UV spectroscopy at 280 nm with molar extinction coefficient values and molecular masses of the proteins as described previously [30, 31].

### Iron oxide nanoparticle preparation

A solution of ferric chloride and ferrous chloride (FeCl_2_ x 4 H_2_O and FeCl_3_ x 6 H_2_O, Sigma, St. Louis, MO) at a 2:1 molar ratio was prepared in deionized water in a round bottom flask. A concentrated ammonia hydroxide solution was added, and the whole solution was stirred for 60 min at 90°C. The total volume of the mixture was 200 mL. After the system reached a precipitation state, it was allowed to cool, and the IONPs settled at the bottom of the flask. The magnetite precipitate was then separated with a neodymium magnet and re-dispersed in fresh deionized water. The magnetite nanoparticles were washed with distilled water three times and dried in an oven at 50°C for 12 h. The dried nanoparticles were re-dispersed in distilled water. To avoid the formation of both maghemite and hematite, the synthesis was performed under oxygen-free conditions [38, 39].

### Transmission Electron Microscopy (TEM)

The structural characterization was carried out on Philips CM 120kV transmission electron microscope equipped with a high-resolution pole piece and CCD camera for image registration at Helmholz Zentrum Berlin. The samples were adsorbed on copper grids covered by amorphous thin film. After drying, it was deposited in TEM and analyzed in bright as well as in diffraction mode.

### Formation of spider silk/iron oxide spheres

The MS1/IONP, MS2/IONP and EMS2/IONP spheres were produced by salting out with a potassium phosphate solution. A 50 μL aliquot of silk protein at a concentration of 5 mg/mL was added to 50 μL of an iron oxide nanoparticle suspension at a concentration of 10 mg/mL, and then, the solution was rapidly mixed with 1000 μL of 2 M potassium phosphate buffer (pH of 8) and incubated overnight. Next, the sphere suspension was centrifuged at 10,000 x g for 20 min, and the spheres were rinsed with ultrapure water. The washing was repeated five times. The MS1/IONP, MS2/IONP and EMS2/IONP spheres suspended in ultrapure water were subsequently sonicated and passed through a PD-10 desalting column (GE Healthcare, Little Chalfont, UK) to remove unbound iron oxide nanoparticles. Control spheres were produced by adding 50 μL of the silk protein at a concentration of 5 mg/mL to 50 μL of 10 mM Tris-HCl (pH of 7.5) and mixing with 1000 μL of 2 M potassium phosphate buffer (pH of 8). To study the properties of EMS2/IONP, the spheres were formed as follows: 50 μL of silk protein at a concentration of 2 mg/mL was added to 50 μL of an iron oxide nanoparticle suspension at a concentration of 10 mg/mL and mixed with 1000 μL of 2 M potassium phosphate buffer at a pH of 8. Control spheres were prepared as described above using a 50 μL of 10 mM Tris-HCl, (pH of 7.5) instead of an iron oxide nanoparticle suspension.

### Scanning Electron Microscopy (SEM) and Energy Dispersive X-ray Spectroscopy (EDXS)

The spheres were sonicated for 5 min in an ultrasonic water bath, and 3 μL of the sphere suspension was placed on a cover glass to dry. The samples were subsequently sputtered with an Au layer using a Quorum Sputter Coater Q150T ES (Quorum Technologies, Ringmer, UK), and then, the samples were analyzed under a JEOL JSM-7001F (JEOL. Ltd, Tokyo, Japan) field emission scanning electron microscope at a 15 kV accelerating voltage. Energy-dispersive X-ray spectroscopy was performed using an X-Max silicon drift detector (Oxford Instruments, Abingdon, UK) and analyzed using INCA Energy software (Oxford Instruments Analytical, High Wycombe, UK).

At least 70 individual spheres were measured on several randomly selected SEM images to calculate an average particle size. The particle sizes were determined with ImageJ 1.46r software by measuring the diameter of separated particles. The values represent the mean from three independent experiments.

### Microwave-Induced Plasma with Optical Emission Spectrometry (MIP–OES)

The samples were analyzed with a Carl Zeiss Echelle spectrometer (Carl Zeiss AG, Oberkochen, Germany) (Model PLASMAQUANT 100) with fiber-optical light-guide, photomultiplier tubes (PMTs) and a TE_101_ microwave plasma cavity. EMS2 and EMS2/IONP spheres were placed in a quartz vessel and dissolved in 0.5 mL of concentrated HNO_3_ and 0.5 mL of 30% HCl. Then, the samples were sonicated at 60% of the ultrasonic probe power setting (ca. 40 W) for 120 s and calibrated to 2 mL using volumetric calibrated flasks. Iron concentrations were analyzed in the continuous mode using an ultrasonic nebulizer (model NOVA-DUO, Optolab, Warsaw, Poland) equipped with a glass cyclonic spray chamber. The experiments were repeated three times.

### Zeta potential

The zeta potential (ZP) was measured using a Zetasizer Nano Z (Malvern Instruments Ltd, Worcestershire, UK). The spheres were suspended in 800 μL of water, sonicated for 5 min in an ultrasonic water bath directly before the measurement and loaded into a capillary cell. The experiment was repeated three times at room temperature.

### Fourier Transform Infrared Spectroscopy (FTIR)

EMS2 and EMS2/IONP spheres were lyophilized, mixed with potassium bromide and then the blended powder was pressed under pressure to form a tablet-shape pellet. The pellets were analyzed with a Bruker Equinox 55/S FTIR spectrometer (Bruker Corp, Fremont, CA). Absorption spectra were obtained from the average of 512 scans with a resolution of 2 cm^-1^ within a wavenumber range of 400 to 4000 cm^-1^. The analysis of spectra was performed with Opus 5.0 software (Bruker Corp. Fremont, CA). The elements of secondary structure were assigned to the amide I band components: 1605–1615 cm^-1^ tyrosine side chains, 1616–1637 cm^-1^ and 1697–1705 cm^-1^ –beta sheets, 1638–1655 cm^-1^ random coils, 1665–1662 cm^-1^ helices, 1663–1696 cm^-1^ turns, according to previously described data [40]. The analysis was performed 1, 7 and 14 days after spheres preparation. The experiment was repeated three times.

### Superconducting Quantum Interference Device (SQUID)

Zero-field-cooled (ZFC) and field-cooled (FC) magnetization and magnetization loops (M vs. H) were performed using a SQUID magnetometer MPMS Quantum Design (Quantum Design International, San Diego, CA). Magnetization loops measurements were carried out at room temperature and 5 K with a magnetic field in the range of ±50 kOe, which was large enough to saturate the magnetization. The temperature dependence of the magnetization was measured at an applied field of 100 Oe between 5 and 350 K.

### Doxorubicin loading and release

EMS2 and EMS2/IONP spheres were loaded with doxorubicin (Adriamycin, Pfizer Inc., New York City, NY) using the post-loading method as described previously [31]. Briefly, 250 μg of spheres were suspended in 250 μL of PBS, mixed with 50 μL of 2 mg/mL doxorubicin and incubated overnight with agitation. The spheres were subsequently centrifuged (15 min, 10 000 x g) and the absorbance of the supernatant was measured at 508 nm to define the drug concentration. The quantification was based on a standard calibration curve of doxorubicin. The loading efficiency was determined using the equation: loading efficiency (%) = (the amount of drug loaded) / (amount of drug before loading) x 100%. For release study, the spheres were incubated in phosphate buffer solution at pH 7.4, 6 and 4.5 at 37°C with agitation. The supernatant was collected at indicated time points and replaced with fresh PBS of proper pH values. The amount of released drug was determined spectrophotometricaly as described above.

### Cytotoxicity

NIH3T3 fibroblasts [31] were cultured in DMEM (Sigma, St. Louis, MO) medium with 10% fetal bovine serum (PAA Laboratories GmbH, Pasching, Austria) and 80 μg/mL gentamycin (KRKA, Novo Mesto, Slovenia) at 37°C in a humidified atmosphere containing 5% CO_2_. A 2.5 x 10^4^/well of cells were seeded onto 96-well microplate and incubated overnight. The next day different concentrations of EMS2 and EMS2/IONP spheres were added to cell cultures. After 72 h of incubation 50 μL of 5 mg/mL MTT reagent (3-(4,5dimethylthiazol-2-yl)-2,5-diphenyl tetrazolium bromide; Sigma, St. Louis, MO) was added to each well and incubated for 4 h. The medium was discarded, and purple formazan was dissolved in 200 μL DMSO (dimethylsulfoxide; Sigma, St.Louis, MO). The absorbance of the solution was measured at a wavelength of 560 nm using Victor X3 microplate reader (PerkinElmer, Waltham, MA). The experiment was repeated three times in triplicates.

### Statistics

The statistical significance of the differences between the sphere variants was calculated using analysis of variance (ANOVA). Post hoc tests with the Bonferroni correction were performed. The differences in the loading efficiency were tested using an unpaired t-test. The differences between groups were considered significant if the *p*-value was less than 0.05.

## Results and discussion

### Iron oxide nanoparticle preparation

Magnetite nanoparticles can be synthesized using various methods, such as ultrasound irradiation [41], sol-gel transformation [42], thermal decomposition [43, 44], or co-precipitation [38]. The most commonly used methods are the last two. Thermal decomposition is based on the pyrolysis of organic iron precursors, which can be Fe(CO)_5_ or Fe(acac)_3_. However, both of these compounds are toxic and may restrict the medical applications of the obtained magnetite. The co-precipitation method is characterized by a simple production and implementation and requires fewer hazardous materials. This method also allows a large number of nanoparticles to be obtained [9]. Moreover, studies on co-precipitated Fe_3_O_4_ nanoparticles have demonstrated their crystalline nature, which is identical to that of magnetite crystals and superparamagnetic properties [38, 39].

**Fig 1:**
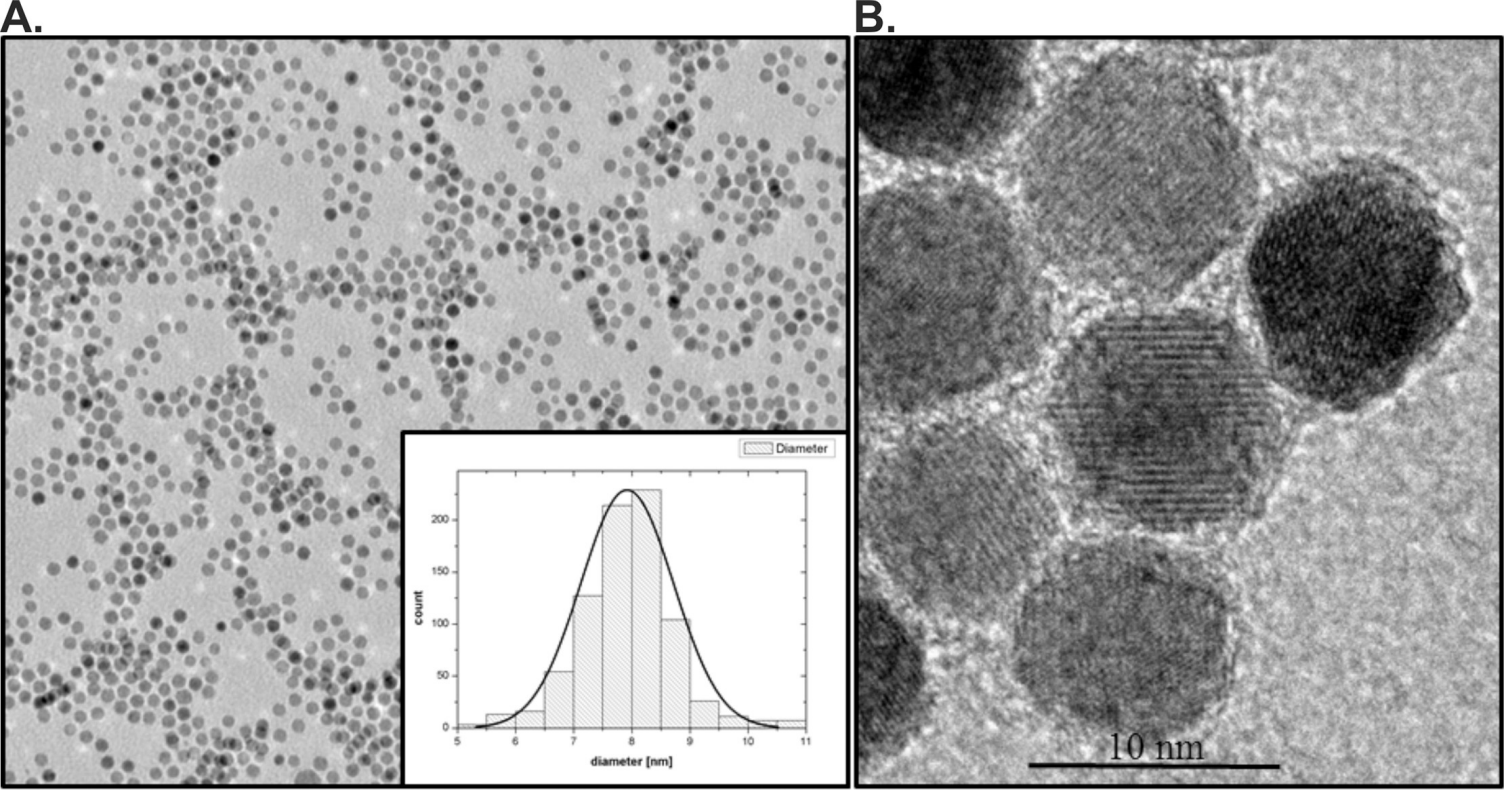
TEM characterization of iron oxide nanoparticles. A) TEM image by low magnification with inserted size distribution of IONPs, B) HRTEM image that shows the ultrastructure and morphology of Fe_3_O_4_ nanoparticles.

For this study, the magnetite nanoparticles (Fe_3_O_4_) were synthesized using a co-precipitation method [38]. The method was based on the chemical reactions in the overall equation: 2Fe^3+^ + Fe^2+^ + 8OH^-^ = Fe_3_O_4_ + 4H_2_O, which was proposed for the mechanism of magnetite formation. Magnetic particles prepared by the described method were stabilized by charge (here negatively). The ammonium cation was located in a second shell around the negatively charged particle surface, thus forming the electrical double layer. The typical TEM image of the IONPs indicated that the morphology of the nanoparticles was spherical and the crystalline structure was confirmed (Fig 1). The superparamagnetic nanoparticles were very monodisperse (SD < 10%) as shown in the TEM image (Fig 1).

### Production and purification of the bioengineered spider silk proteins MS1, MS2, EMS2

Three bioengineered spider silks were used in the study: MS1, MS2, and EMS2. The MS1, MS2, and EMS2 proteins differ in their amino acid sequence, which results in their different properties such as their hydrophobicity, isoelectric point (pI) and charge at an indicated pH (summarized in Table 1). These properties can affect the interactions between silk and iron oxide nanoparticles during the sphere formation process. MS1, MS2, and EMS2 proteins were produced in *E*.*coli* and purified from bacterial cells using a thermal denaturation method as described previously [5, 30, 31, 37]. The yield of the purification was 1.8, 2 and 3 mg from 1 g of bacterial pellet for the MS1, MS2, and EMS2 silks, respectively. The purified proteins had good quality and purity, and no impurities and protein degradation were observed in the SDS-PAGE gel, what was indicated in our previous studies [30, 31].

**Table 1:** The properties of the bioengineered spider silk proteins MS1, MS2 and EMS2 predicted on the basis of their amino acid sequences.

	MS1	MS2	EMS2
**Hydropathicity** **(GRAVY)**	-0.139	-0.535	-0.613
**Isoelectric point** **(pI)**	10.83	5.27	3.15
**Charge at pH 8**	(+)	(-)	(-)

### Morphology of the spider silk/iron oxide spheres

Spheres were formed upon mixing a high concentration of the potassium phosphate buffer with a silk solution or with a silk solution and iron oxide nanoparticles. The salting out method was previously used successfully to produce spheres made of MS1, MS2, EMS2, eADF4(C16) bioengineered spider silk [45, 46] and silkworm silk fibroin [4, 28]. The eADF4(C16) silk was based on the dragline silk protein ADF4 from the *Araneus diadematus* spider [45].

**Fig 2:**
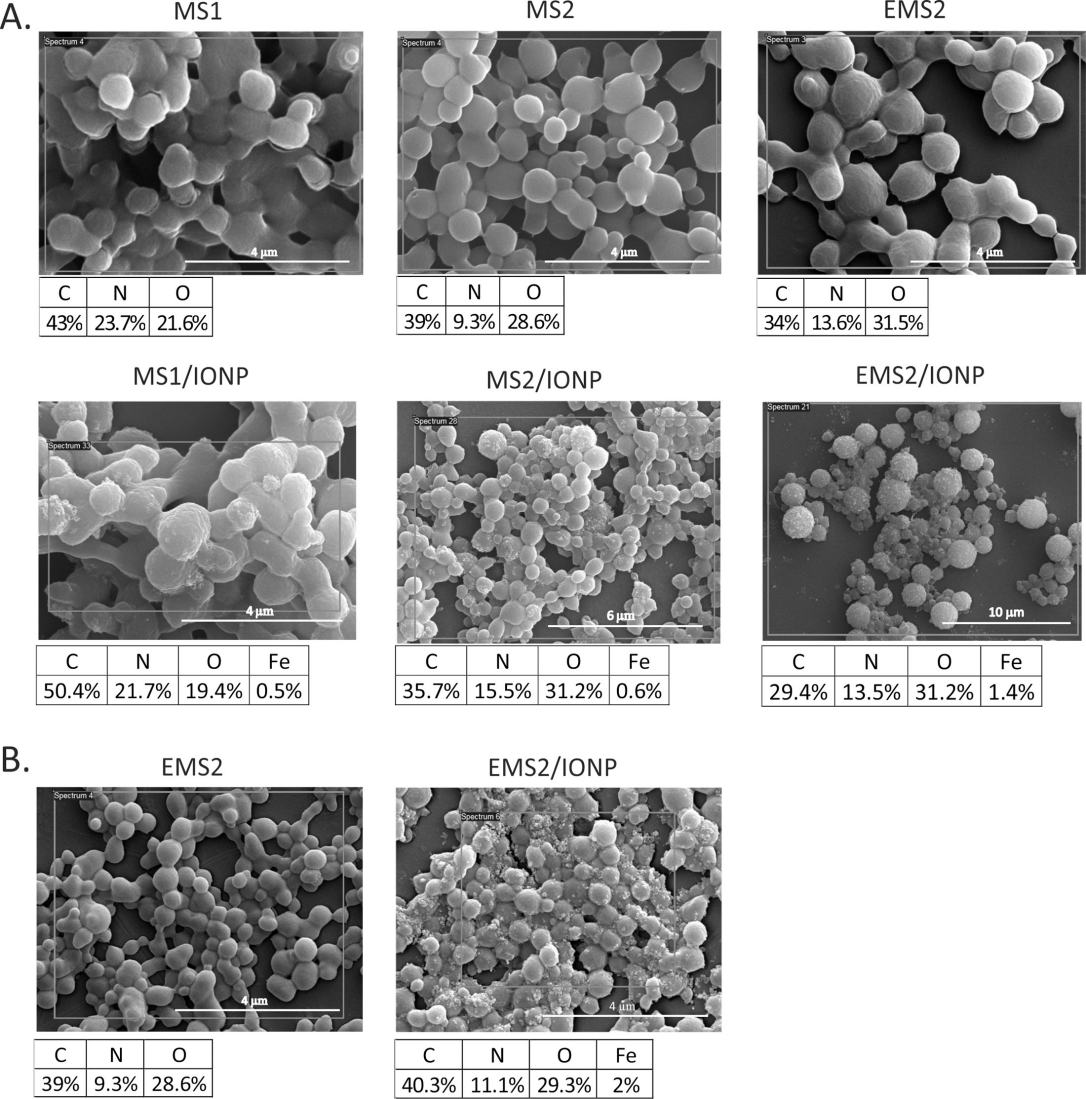
SEM images and EDXS quantitative results of selected elements of MS1, MS1/IONP spheres, MS2, MS2/IONP spheres, EMS2, and EMS2/IONP spheres. A) Spheres were prepared by mixing 2 M potassium phosphate at pH 8 with the indicated version of silk (2.5 mg/mL) in the presence or absence of iron oxide nanoparticles (5 mg/mL). B) EMS2 and EMS2/IONP spheres were formed by mixing 2 M potassium phosphate at pH 8 with a silk solution (1 mg/mL) in the presence or absence of the iron oxide nanoparticles (5 mg/mL); scale bar– 4 μm.

The presence of iron oxide nanoparticles did not impede silk self-assembly to form spheres (Fig 2). The morphologies of the spheres made of plain silks and silk/iron oxide composites were different (Fig 2). Moreover, the plain silk spheres presented various morphologies depending on the protein used (Fig 2A), which was investigated previously [30, 31]. As mentioned above, these silks differed in their amino acid sequences, which determine their properties (Table 1). It was shown previously that the MS2 protein polymerizes more rapidly than MS1 to form smooth, spherical particles with a lower tendency to aggregate. The morphology of the MS2 spheres could be a result of a higher β-sheet content than the MS1 spheres [30]. The EMS2 protein was obtained by modifying MS2 by adding a hydrophilic glutamic acid residue to each MS2 repeating unit. The morphologies of the MS2 and EMS2 spheres were different, although both types of spheres presented the same secondary structure content. According to the GRAVY index, EMS2 silk is more hydrophilic than the MS2 protein, which could result in the different, stickier morphologies of the EMS2 spheres. This phenomenon might be due to the various assembly processes of these silks [31]. The AFM analysis previously indicated that both EMS2 and MS2 spheres were round, of smooth surfaces and revealed good structural integrity, even if they formed clusters [31]. The size and aggregation behavior of silk spheres is an important aspect of the characteristics of drug carriers and it needs further study. In the future, the dynamic light scattering (DLS) may introduce important data.

The iron oxide nanoparticles bound to all the MS1, MS2 and EMS2 spheres. MS1/IONP, MS2/IONP, and EMS2/IONP carriers aggregated more than the plain MS1, MS2, and EMS2 silk spheres, respectively. However, they preserved a spherical morphology (Fig 2). The IONPs tended to gather in tight clusters and were found on the surface of the spheres in the form of small bumps, as observed in the SEM image (Fig 2). For the MS1/IONP and MS2/IONP spheres, the IONPs clusters were located at random areas, whereas the EMS2/IONP spheres were more uniformly covered with IONPs. The presence of magnetite nanoparticles in the obtained spheres was confirmed by the elemental analysis using EDXS (Fig 2). The results showed that the MS1/IONP, MS2/IONP, and EMS2/IONP carriers contained iron oxide nanoparticles. The highest IONPs content was detected in the EMS2/IONP spheres (Fig 2). Zhang et al. demonstrated that Fe_3_O_4_ and silkworm silk formed composite spheres in the process of salting out by using a potassium phosphate buffer [4]. On the surface of these spheres, aggregates of Fe_3_O_4_ nanoparticles were observed with TEM microscopy. It was proposed that a high concentration of ions (potassium phosphate) can prevent the accumulation of the negatively charged iron oxide nanoparticles inside the obtained carriers [4]. The same could apply to our EMS2/IONP spheres.

### Size of the spider silk/iron oxide spheres

**Fig 3:**
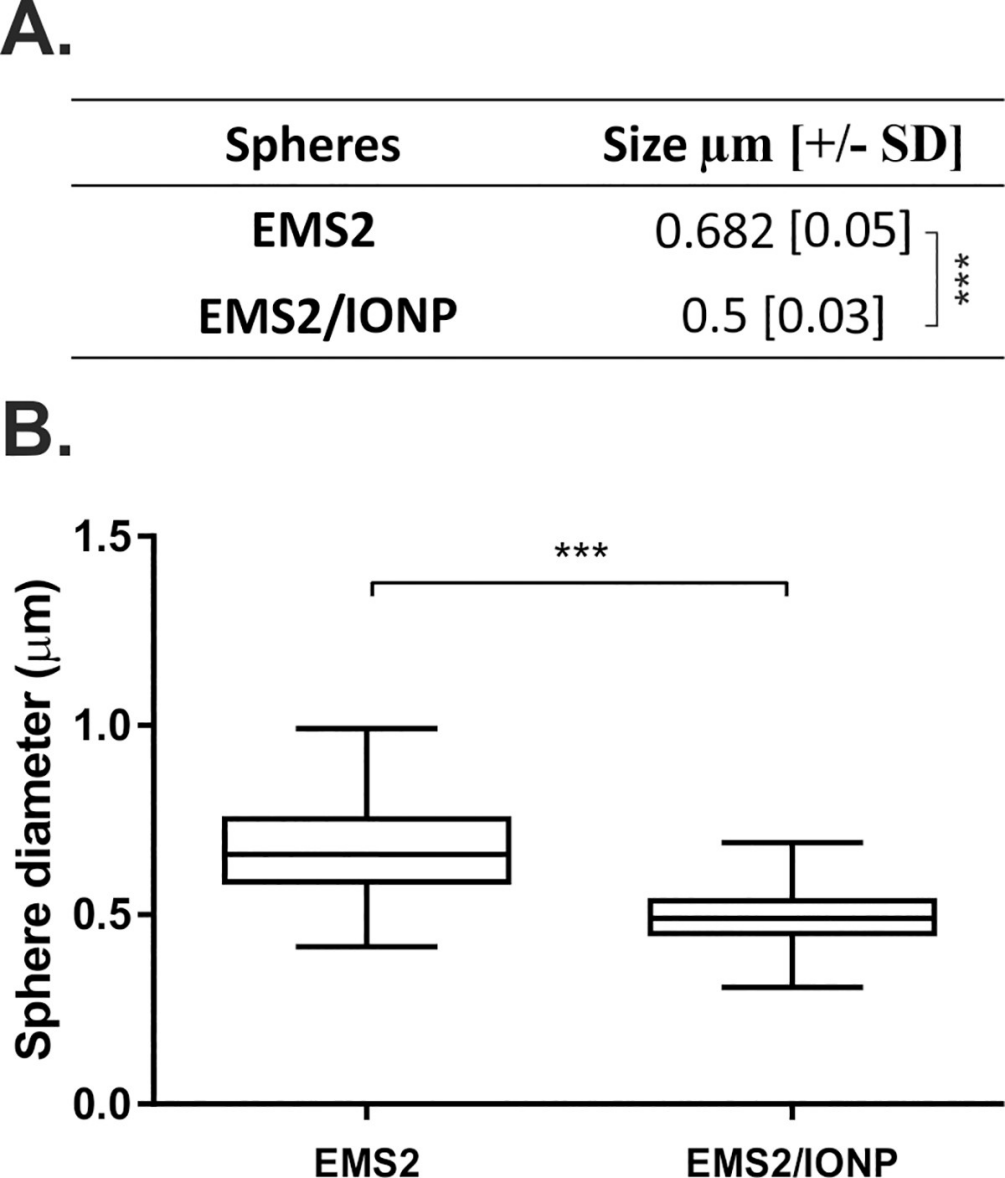
Size analysis of the EMS2 and EMS2/IONP spheres. For the A) size and B) size distribution analyses, the spheres were prepared by mixing a silk solution (1 mg/mL) and iron oxide nanoparticles (5 mg/mL) with 2 M potassium phosphate at pH 8. B) Whiskers show the minimal and maximal sizes of the spheres, and the line inside the box shows the median size. ***indicates statistical significance with p < 0.0001.

Based on the obtained results, we chose EMS2/IONP composite particles for further detailed analysis. The size of the nanoparticles used as carriers plays an important role in their pharmacokinetics. Larger particles more efficiently activate the human complement system and are removed more quickly from organisms than their smaller equivalents [47]. We and others have previously demonstrated the correlation between the initial silk concentration and size of the formed spheres [28, 31, 48, 49]. Therefore, to obtain smaller and more homogeneous particles, we reduced the initial silk concentration before the sphere formation. The spheres formed by mixing silk at a concentration of 1 mg/mL with a potassium phosphate buffer had a spherical morphology with both EMS2 and EMS2/IONP (Fig 2B). As expected, the spheres prepared from an initial silk concentration of 2.5 mg/mL were larger than those obtained from silk at a concentration of 1 mg/mL (Fig 2A vs. 2B). Moreover, we observed a reduction in the mean size of the spheres that formed in the presence of iron oxide nanoparticles compared with the spheres made of plain silk (Fig 3A). Furthermore, the EMS2/IONP particles exhibited a narrower size distribution than the EMS2 spheres (Fig 3B). The decrease in the sphere size might be due to the higher concentration of the initial solution of silk/iron oxide compared to that of the plain silk prior to the sphere formation. The studies by Chen et al. demonstrated that the addition of methotrexate to a silk fibroin solution and Fe_3_O_4_ suspension resulted in the formation of smaller spheres compared with those prepared by mixing silk and iron oxide nanoparticles [36]. Tu et al. reported that such an observation follows the conventional crystallization theory, which is based on the idea of solution supersaturation levels being a force for precipitation [50]. Higher concentration solutions can reach supersaturation faster than a less concentrated solution, which results in an increased nucleation rate, and smaller particles precipitate from a more concentrated solution [50].

### Secondary structure analysis of plain and composite spheres

**Fig 4:**
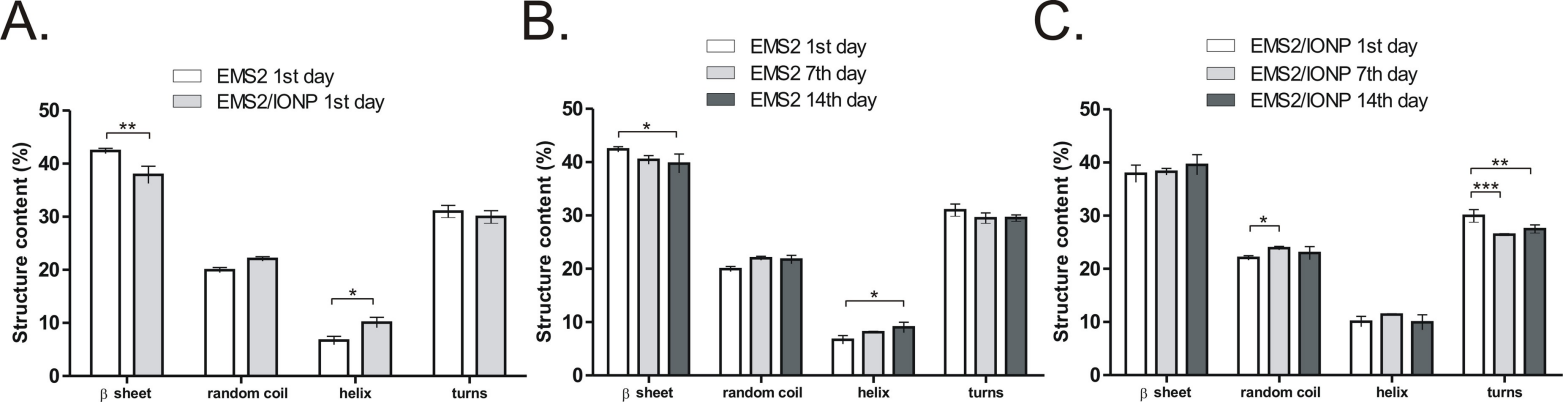
Secondary structure analysis of the EMS2 and EMS2/IONP spheres. A) The secondary structure composition of the EMS2 and EMS2/IONP spheres investigated on the 1^st^ day of storage. B, C) Analysis of the secondary structure composition of the (B) EMS2 and (C) EMS2/IONP spheres during storage on the 1^st^, 7^th^ and 14^th^ day. The secondary structure content of both types of particles was calculated after Fourier self-deconvolution of the amide I region of the FTIR spectra. The mean and error bars indicating the standard deviations are shown. The experiment was repeated three times. * indicates statistical significance with: * p < 0.05; ** p < 0.01; *** p < 0.001.

The secondary structure composition of the EMS2 and EMS2/IONP spheres was estimated using the Fourier deconvoluted infrared spectra of the amide I band analysis. The IONPs caused a significant reduction in the beta-sheet content and an increase in the random coil and helices contents in the examined spheres (Fig 4A). However, the beta-sheet structures were dominant in both the EMS2 and EMS2/IONP spheres, which indicated the preservation of the silk sphere crystalline structure in the presence of magnetite.

The primary amino acid sequence of spider silk proteins is responsible for their extraordinary physical properties. The stability and strength of silk materials are the result of the presence of polyalanine motifs (poly(A)), which form beta-sheet structures [51]. Therefore, it is necessary to preserve silk protein secondary structures while combining them with various compounds to prepare a composite material. The studies of Zhang et al. observed that both silk fibroin and silk fibroin/iron oxide nanoparticles exhibited mostly beta-sheet structures, which indicated that the Fe_3_O_4_ NPs did not have a major impact on the silk crystalline structure [4]. Ma et al. showed the negligible influence of PEG and PLGA compounds on the structural analysis of the silk hydrogel composite [52]. However, there are examples that the silk secondary structure changed due to the addition of another element during the formation of a composite material [53]. Feng et al. demonstrated that a film made of plain silk fibroin consisted mostly of random-coil and alpha-helix structures, while TiO_2_/silk fibroin composite films exhibited mostly beta-sheet content [53].

The structure analysis study was performed at three measurement points, i.e., after 1, 7 and 14 days of storage of the spheres at 4°C in water. We observed some fluctuations in the content of the secondary structures of spheres up to the 14^th^ day of storage (some differences were significant). From the other hand, previously we analyzed the morphology of plain EMS2 spheres for six months of storage at 4°C in water and we did not observe any visible modification in their spherical morphology [31]. However, these data were qualitative but not quantitative. It may be interesting to analyze the spheres after a longer period of storage in the terms not only of the morphology of the spheres but also regarding secondary structure and quantity of spheres, and, in case of composite spheres, regarding the content of iron oxide. It needs further evaluation.

### Zeta potential of spheres

The zeta potential measurements confirmed the presence of IONPs on the surface of the EMS2/IONP spheres. Both the EMS2 spheres and IONPs demonstrated negative zeta potential values (Table 2). However, the presence of magnetite nanoparticles on the sphere surfaces resulted in a significant reduction in the ZP value (closer to 0 mV) of the EMS2/IONP spheres (Table 2). Since the IONPs “formed small bumps” and did not evenly cover the entire surface of the spheres, the ZP value of composite spheres can be an intermediate value between ZP of IONPs and EMS2 spheres. Based on these data, we concluded that electrostatic interactions played a minor role in combining silk with IONPs. Previous studies demonstrated the possibility of binding different components with the same (positive or negative) charge to silk proteins. This phenomenon was observed for positively charged MS1 silk carriers loaded with positive Dox [5] and positively charged eADF4 silk spheres with positively charged FITC-lysozyme and FITC-BSA [54].

**Table 2:** The iron content and zeta potential of the spheres.

	ZP mV [+/- SD]	Iron content (μg Fe/μg spheres)
**EMS2**	-38.1 [1.1]	0.0
**EMS2/IONPs**	-31.97 [0.6]**	0.029
**IONPs**	-25.3 [0.9]	ND

The spheres were produced by mixing 2 M potassium phosphate at a pH of 8 with silk (1 mg/mL) in the presence or absence of iron oxide nanoparticles (5 mg/mL). The results of the zeta potential show the mean value and SD of three experiments in triplicate.

** indicates statistical significance with p < 0.01

### Iron oxide nanoparticle content

EMS2 and EMS2/NP spheres were analyzed to determine the amount of iron nanoparticles. The microwave-induced plasma with optical emission spectrometry demonstrated the absence of iron ions in the EMS2 spheres (Table 2). The concentration of iron ions in the sample of EMS2/IONP spheres was 2.3 μg/mL (+/- 0.16), which after recalculation resulted in 0.029 μg Fe^3+^ per 1 μg of spheres (approximately 2.9% IONPs content). This analysis was in agreement with the results obtained using EDXS (Fig 2).

### Magnetic properties of the IONPs and composite spheres

**Fig 5:**
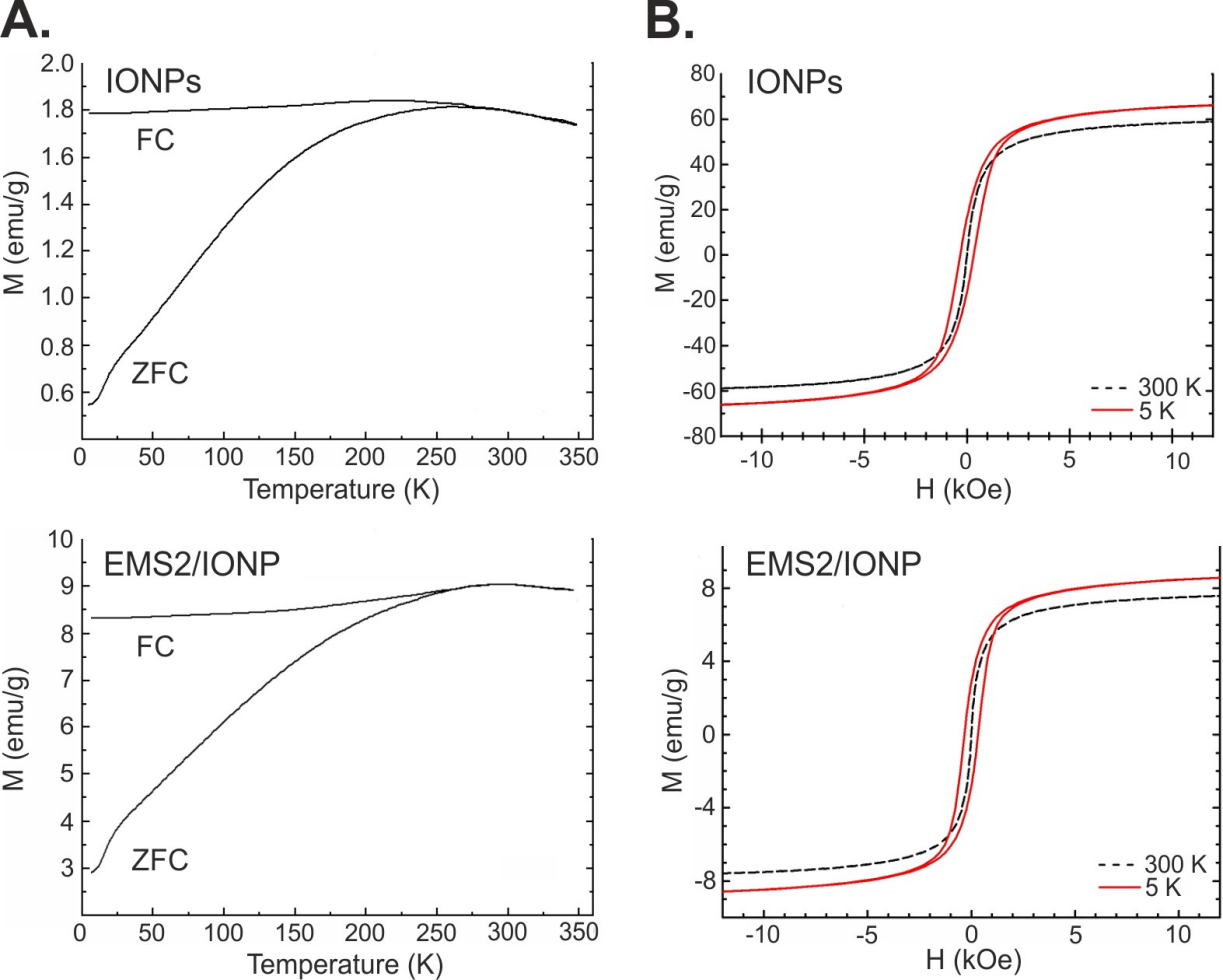
Magnetic measurements of the IONPs and silk/ iron oxide spheres (EMS2/IONP). A) Zero-field-cooled (ZFC) and field-cooled (FC) magnetization curves for IONPs (upper) and EMS2/IONP spheres (bottom); B) Hysteresis loops at T = 5 and 300 K for IONPs (upper) and EMS2/IONP (bottom) spheres.

The magnetic properties of the EMS2/IONP spheres were analyzed and compared to those of the plain magnetite nanoparticles using a superconducting quantum interference device. Fig 5 shows the magnetization curves of the IONPs measured before and after loading on EMS2 spheres. The ZFC curves of all the nanoparticles showed typical peak features at certain temperatures, i.e., the so-called blocking temperature, T_*B*_, at 298 K and 262 K for the IONP and EMS2/IONP samples, respectively. This decrease in *T*_*B*_ indicated that the interparticle interactions were weakened in the presence of silk, which was probably due to an increase in the average distance between the nanoparticle cores. Similar decreases in *T*_*B*_ were previously reported with the increasing interparticle distance, which was accomplished by increasing the particle concentration in a suspension [55, 56]. The theoretical simulations [57] as well as experimental studies [58] on superparamagnetic nanoparticles have also shown that the blocking temperature is proportional to the inverse cube of the interparticle spacing, *T*_*B*_ ~ d^−3^.

Above the blocking temperature, the FC and ZFC curves exhibited similar behaviors, which indicated the nanoparticles were free to align with the field at high temperature. This state can be considered superparamagnetic. At low temperatures below the *T*_*B*_, the FC curves decreased slightly and finally plateaued. A similar behavior was already observed in other systems [58–61], and in our case, the behavior can be explained considering that magnetite, which was the main component in the samples, is ferrimagnetic. At low temperatures, ferrimagnetic coupling within particle cores is stronger than the weak external field and forces the magnetic moments into the antiparallel or tilted configuration, which reduces the total magnetic moment. At higher temperatures, the ferrimagnetic coupling weakens, and simultaneously, thermal fluctuations become increasingly significant. Magnetic moments gain more freedom and are more likely to arrange along the lines of an external magnetic field, which increases the total magnetization. At temperatures higher than *T*_*B*_, the thermal fluctuations are large enough to disorder the system, leading to a reduction in the measured magnetic moment.

Fig 5B shows the hysteresis loops at 5 and 300 K for iron oxide nanoparticles and EMS2/IONP spheres. Both samples at room temperature showed the expected behavior and did not display magnetic remanence nor a coercive field. The initial slopes of the magnetization curve were steep, and the magnetic susceptibility near *H* = 0 was 9 x 10^−2^ emu x Oe^-1^ x g^-1^ and 2 x 10^−2^ emu x Oe^-1^ x g^-1^ for the IONPs and EMS2/IONP spheres, respectively. At 300 K, the nanoparticles can be considered superparamagnetic; however, at 5 K, the hysteresis loops for both samples showed ferromagnetic properties. The coercive field, *H*_*c*_, in both cases was approximately 330 Oe, while the remanent magnetization, *M*_*r*_, was approximately 16.3 emu x g^-1^ and 2.8 emu x g^-1^ for the IONPs and EMS2/IONP spheres, respectively. The saturation magnetization, *M*_*s*_, at 5 K for the IONP sample was approximately 70 emu x g^-1^, which was smaller than that of the bulk magnetite but agreed with the data for magnetite nanoparticles reported in the literature [60]. The differences may be due to dipolar interactions among the nanoparticles, different particle sizes or spacings among the particles, or the presence of admixtures of other iron oxides, such as hematite. The value of the saturation magnetization of the sample EMS2/IONP was much smaller than that of the IONP sample, and this was a direct result of the fact that a significant proportion of the sample mass was non-magnetic silk, which did not add to the magnetic moment.

### Drug loading and release

**Fig 6:**
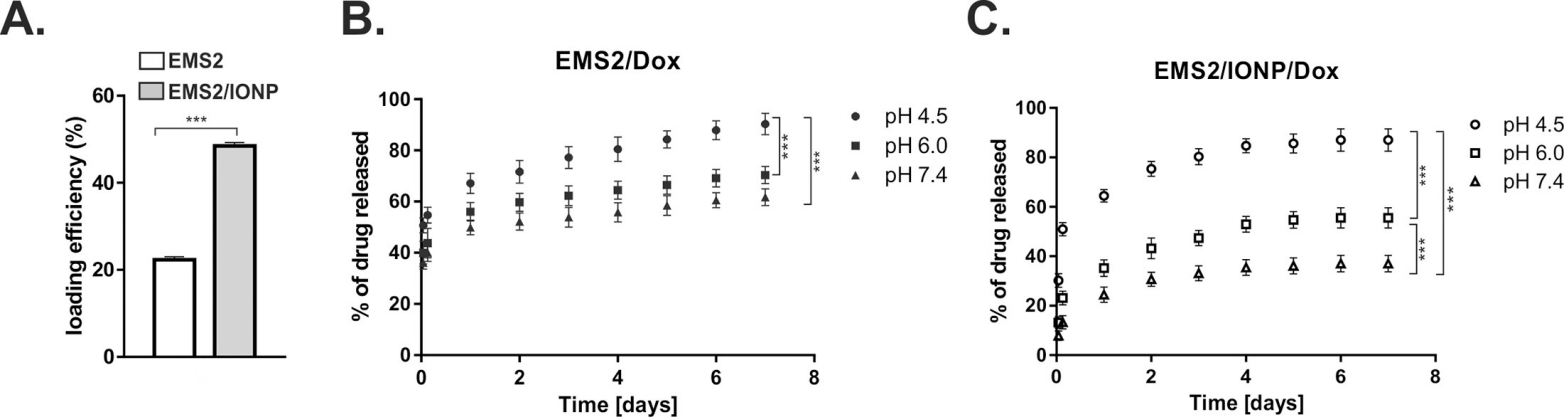
The loading efficiency and release kinetics of doxorubicin (Dox). A) Spheres were loaded with Dox using a post-loading method. B, C) From the B) EMS2 and C) EMS2/IONP spheres, doxorubicin was released at 37°C in PBS buffers at pH values of 7.4, 6 and 4.5 over 7 days. The time points for the drug release measurements were after 1 and 3 h of incubation on day 1 and then every 24 h. The means and standard deviations of three independent experiments are shown. * indicates statistical significance with p < 0.0001; *** indicates statistical significance with p < 0.001.

**Fig 7:**
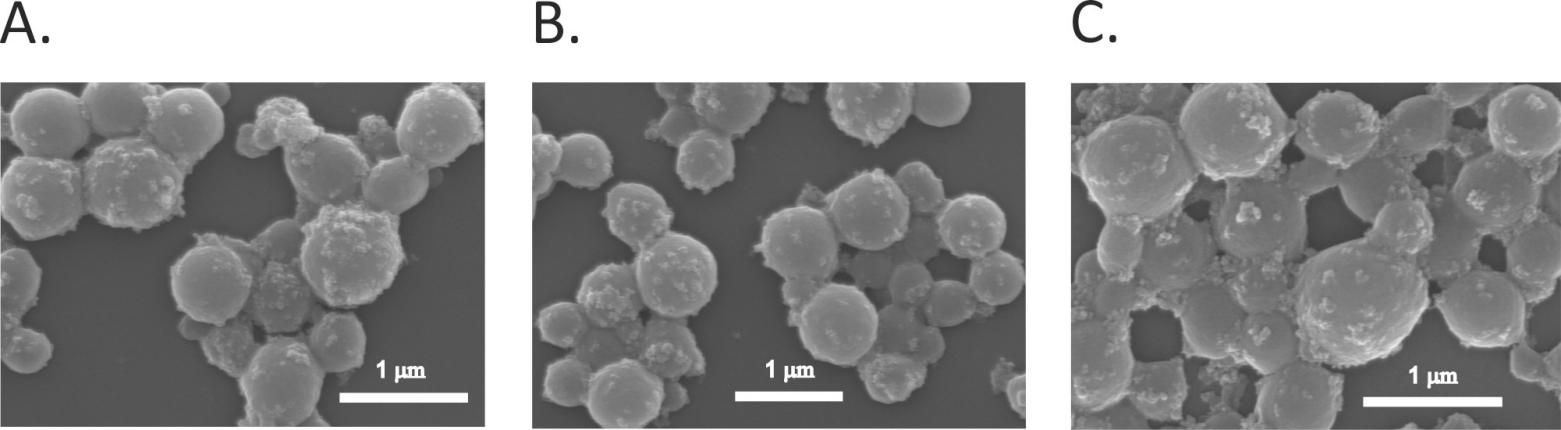
The SEM images of A) EMS2/IONP spheres B) EMS2/IONP spheres loaded with doxorubicin and C) EMS2/IONP spheres after the release of doxorubicin. EMS2/IONP spheres were prepared by mixing 2 M potassium phosphate at pH 8 with silk (1 mg/mL) and iron oxide nanoparticles (5 mg/mL) and then were loaded with Dox by the post-loading method. Next, Dox was released from EMS2/IONP spheres for 7 days at pH of 4.5; scale bar– 1 μm.

Drug loading was performed using a post-loading method, as described previously.[30] For both EMS2 and EMS2/IONP spheres, a total of 250 μg of particles was incubated with doxorubicin. The loading efficiency of Dox into the EMS2 spheres was similar to that reported previously (Fig 6A) [31]. For the EMS2/IONP composite spheres, the loading efficiency of the drug was more than two times higher than that for the plain EMS2 particles (Fig 6A). Thus, we observed a higher drug loading efficiency for positively charged doxorubicin on the less negative EMS2/IONP spheres than on the EMS2 carriers. Various factors could have an impact on the obtained data. As indicated in Figs 2 and 7, IONPs particles were exposed on the surface of the sphere in a form of small bumps and they may constitute an additional binding site for the positively charged drug. Moreover, the difference in the loading efficiency of Dox might be caused by the difference in the size of the examined particles. The same amount (250 μg) of the significantly smaller EMS2/IONP spheres resulted in a larger overall particle surface that could bind more doxorubicin than the EMS2 carriers. Another possibility that needs to be explored is the analysis of the core of EMS2 and EMS/IONP spheres. Previously we showed the difference between MS1 and MS2 spheres in terms of their core and content of the secondary structures [30]. We pointed out the possibility that the looser packing of the MS1 spheres (of significantly lower beta-sheet content) and their inner pores could enable the penetration of drug molecules through the sphere and thus contributing to higher Dox uptake than to the MS2 spheres [30]. From the other hand, the solid, condensed protein core of the MS2 particles (of significantly higher beta-sheet content) could result in lower diffusion-based loading of doxorubicin [30]. The analysis of the secondary structure content of MS2 and EMS2 spheres did not indicate the differences between both spheres (approximately 40% of beta-sheet for both sphere types) [31]. The loading of Dox for MS2 and EMS2 spheres was similar [31]. It may indicate that the core of MS2 and EMS2 may be similar. Since the beta-sheet content in EMS2/IONP spheres was significantly less than in EMS2 spheres, the looser packaged silk molecules could similarly enable more efficiently penetration of doxorubicin. However, this issue needs further study.

A release study of incorporated doxorubicin was carried out at 37°C over a period of 7 days using phosphate buffered saline at pH values of 4.5, 6 and 7.4 (Fig 6B and 6C). For both the EMS2 and EMS2/IONP spheres, the drug demonstrated a pH-dependent release profile with the highest and lowest efficiencies at pH values of 4.5 and 7.4, respectively. Fig 7 shows a similar morphology of the different variants of EMS2/IONP spheres:—plain, loaded with Dox and after the release of Dox. The highest release of Dox at pH of 4.5 probably neither dependent on the degradation of spheres nor decomposition of composite spheres. The results in previous studies revealed that doxorubicin exhibited a pH-dependent release profile from silk fibroin particles [62] and MS1, MS2 and EMS2 spider silk spheres [5, 30, 31]. One possible explanation of the observed results might be the fact that a lower pH increased the hydrophilicity of doxorubicin, resulting in a faster release rate from the carriers [63]. The influence of the pH value on the doxorubicin release profile was reported for the iron oxide/silk fibroin composite spheres as well [4]. However, these studies did not compare the release kinetics of plain silk fibroin spheres and silk fibroin/iron oxide composite particles [4]. In this study, we observed the distinct drug release kinetics from EMS2 and EMS2/IONP spheres (Fig 6B and 6C). The EMS2/IONP spheres released significantly less Dox at pH values of 6 and 7.4 than the plain silk spheres and a comparable amount of Dox at a pH of 4.5 (Fig 6B and 6C). This phenomenon could have a great importance for cancer therapy applications. Our system could enable effective drug transport in the blood (limited release) to a tumor site (enhanced release).

### Cytotoxicity

**Fig 8:**
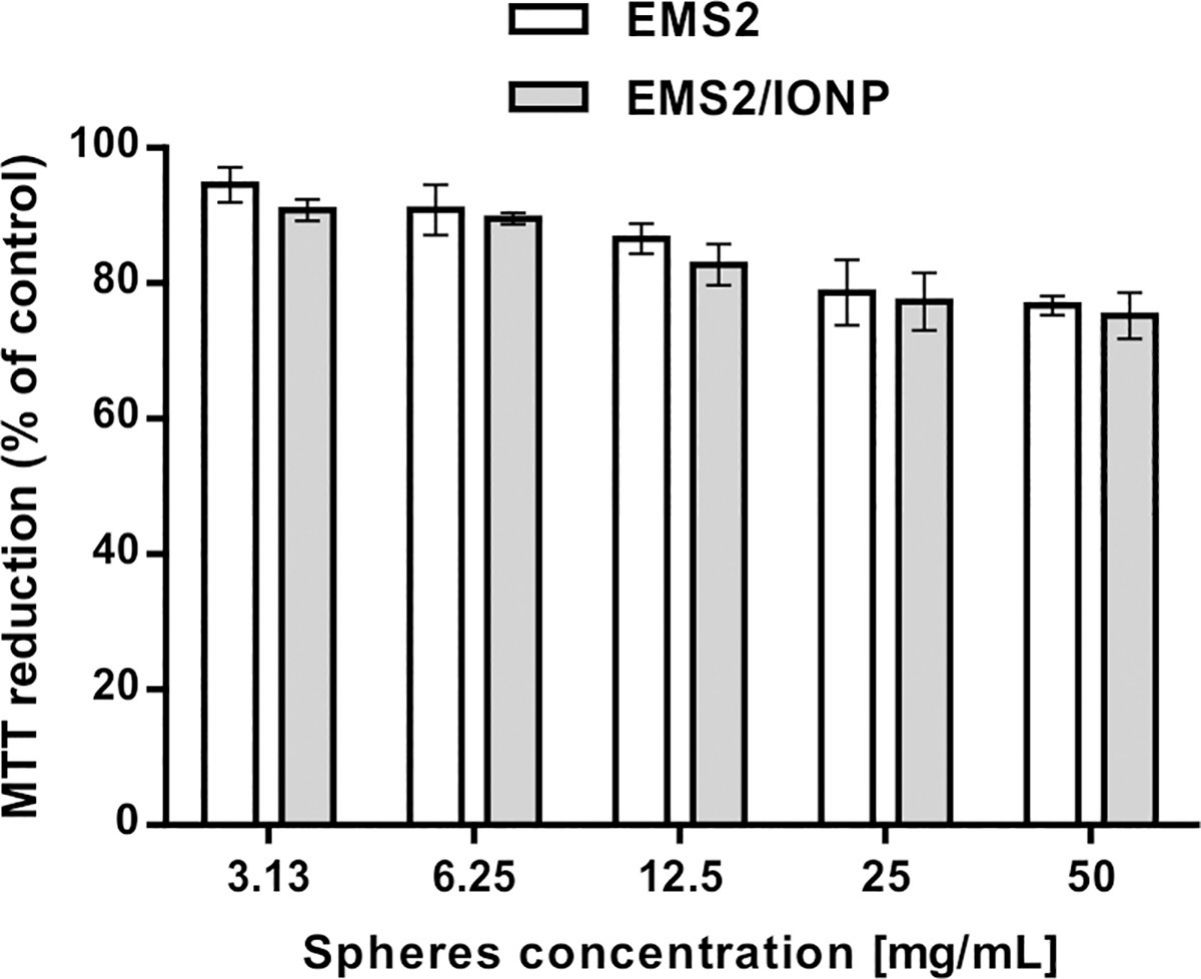
Cytotoxicity study by MTT assay. The NIH3T3 fibroblasts were cultured in the presence of EMS2 and EMS2/IONP spheres for 72 h. Spheres were produced using an initial silk concentration of 1 mg/mL and 2 M potassium phosphate (pH 8) in the presence or absence of IONPs. The MTT reduction was calculated with reference to the non-treated cells. Error bars show the standard deviations of the mean of three independent experiments.

The MTT cytotoxicity study showed that both the EMS2 and EMS2/IONP spheres were not toxic (Fig 8). The presence of iron oxide nanoparticles did not cause an increase in the cytotoxicity of the composite spheres compared to that of the plain silk particles (Fig 8). Similar results were presented in the studies of Zhang et al. [4]; i.e., the iron oxide/silk fibroin composite microspheres exhibited no indication of cytotoxicity towards HeLa cells. Thus, the non-cytotoxic silk spheres covered with IONPs are promising for *in vivo* applications.

## Conclusions

We investigated a composite material made of bioengineered spider silk and iron oxide nanoparticles. Composite spheres were obtained from MS1, MS2, and EMS2 silk variants, and the EMS2/IONP spheres were chosen for further investigation because they had the highest IONP content. The presence of magnetite nanoparticles in the spheres was confirmed using several methods such as SEM, EDXS, MIP-OES and zeta potential measurements. The addition of iron oxide nanoparticles influenced the secondary structure of the spheres; however, beta-sheet structures were dominant in composite particles. Moreover, EMS2/IONP spheres demonstrated good magnetic properties compared to those of the plain magnetite nanoparticles. Furthermore, the composite spheres exhibited a more than two-fold higher loading efficiency for doxorubicin than the plain EMS2 particles. A similar release behavior for the drug was observed from both types of spheres under acidic conditions. However, a slower release was observed from the composite spheres in a neutral pH compared to that of the plain silk spheres. It may be important for drug delivery applications because a chemotherapeutic is released at the tumor site (acidic environment) and not during carrier transport inside the organism. Moreover, the addition of iron oxide nanoparticles to biocompatible silk did not result in the cytotoxicity of the obtained spheres. However, the localization of iron oxide nanoparticles on the surface of the spheres could be a drawback for *in vivo* applications. Further studies are also required to enhance the number of IONPs that are bound on the surface or incorporated inside the silk spheres, which may be important for therapeutic use. We also reported previously that the functionalization of silk with cell binding proteins was necessary to obtain the internalization of spheres in cells [5]. Another approach that could improve the potential of the composite materials could be blending different silks during the sphere formation process [64]. This strategy could combine the most favorable properties of the various silks, which could lead to the formation of more effective tools for cancer therapy.

The results of our study demonstrated the possibility to produce cytocompatible spheres made of iron oxide/spider silk composite materials with a high magnetism and enhanced potential for chemotherapeutic drug loading. Such a vehicle can be potentially applied for magnetic resonance imaging and as a drug carrier for cancer therapy. Moreover, the combined treatment of hyperthermia and drug delivery may enhance the effectiveness of cancer treatments.
